# Evaluating Natural History and Follow Up Strategies for Non-obstructive Urolithiasis in Pediatric Population

**DOI:** 10.3389/fped.2018.00353

**Published:** 2018-11-16

**Authors:** Kunj R. Sheth, Jeffrey T. White, Andre F. Perez-Orozco, Natalie D. Debolske, Christopher R. Hyde, Christine Geistkemper, David R. Roth, Paul F. Austin, Edmond T. Gonzales, Nicolette K. Janzen, Duong D. Tu, Angela G. Mittal, Chester J. Koh, Sheila L. Ryan, Carolina Jorgez, Abhishek Seth

**Affiliations:** ^1^Scott Department of Urology, Baylor College of Medicine, Houston, TX, United States; ^2^Division of Urology, Department of Surgery, Texas Children's Hospital, Houston, TX, United States

**Keywords:** pediatric nephrolithiasis, observation, kidney stones, renal ultrasound, metabolic stone disease, ureteroscopy, percutaneous nephrolithotomy

## Abstract

**Objective:** While small non-obstructive stones in the adult population are usually observed with minimal follow-up, the same guidelines for management in the pediatric population have not been well-studied. We evaluate the clinical outcomes of small non-obstructing kidney stones in the pediatric population to better define the natural history of the disease.

**Methods:** In this IRB-approved retrospective study, patients with a diagnosis of kidney stones from January 2011 to March 2017 were identified using ICD9 and ICD10 codes. Patients with ureteral stones, obstruction, or stones >5 mm in size were excluded. Patients with no follow-up after initial imaging were also excluded. Patients with a history of stones or prior stone interventions were included in our population. Frequency of follow-up ultrasounds while on observation were noted and any ER visits, stone passage episodes, infections, and surgical interventions were documented.

**Results:** Over the 6-year study period, 106 patients with non-obstructing kidney stones were identified. The average age at diagnosis was 12.5 years and the average stone size was 3.6 mm. Average follow-up was 17 months. About half of the patients had spontaneous passage of stones (54/106) at an average time of 13 months after diagnosis. Stone location did not correlate with spontaneous passage rates. Only 6/106 (5.7%) patients required stone surgery with ureteroscopy and/or PCNL at an average time of 12 months after initial diagnosis. The indication for surgery in all 6 cases was pain. 17/106 (16%) patients developed febrile UTIs and a total of 43 ER visits for stone-related issues were noted, but no patients required urgent intervention for an infected obstructing stone. Median interval for follow-up was every 6 months with renal ultrasounds, which then was prolonged to annual follow up in most cases.

**Conclusions:** The observation of pediatric patients with small non-obstructing stones is safe with no episodes of acute obstructive pyelonephritis occurring in these patients. The sole indication for intervention in our patient population was pain, which suggests that routine follow-up ultrasounds may not be necessary for the follow-up of pediatric non-obstructive renal stones ≤5 mm in size.

## Introduction

The incidence of pediatric nephrolithiasis is increasing with recent estimates reporting 57 per 100,000 patients with new stones in 2008 and an annual increase of 4–10.8% per year (1, 2). Furthermore, children with nephrolithiasis have a high recurrence rate of 16–44% (3–6). Although there are several options for management of renal stones (observation, percutaneous nephrolithotomy, shock-wave lithotripsy, and ureteroscopy), observation or watchful waiting has been recommended for asymptomatic renal calculi (7, 8). However, due to low rates of spontaneous passage, both the European Association of Urology and the American Urological Association have size recommendations for stone intervention (7, 8). In children, Telli et al. identified symptoms, stone size progression, stone size of 7 mm or more, accompanying renal anatomical anomalies and cystine or struvite stones as independent predictors of surgical intervention (9).

Few studies have evaluated the long-term outcomes of conservatively managed asymptomatic renal calculi and even fewer in pediatric cohorts. Retrospective studies in pediatric patients to assess spontaneous stone passage rates have shown a 60% passage rate of stones < 5 mm for all ages (5, 6, 10, 11). AUA recommendations suggest that uncomplicated pediatric stones < 10 mm can be managed with observation with periodic ultrasonography (7). Nonetheless, there is little information regarding the long-term outcomes of such patients with stones ≤5 mm in size, especially since up to 44% of patients will have repeat stone episodes. To determine whether prophylactic intervention or long-term follow-up is necessary for this group of patients, the natural history of asymptomatic stones ≤5 mm in pediatric patients must be better understood.

Dos Santos et al. have reported on conservative management in pediatric patients with lower pole asymptomatic stones ≤5 mm in size; out of 224 children, ~54% passed stones, 25% remained asymptomatic and ~21% required intervention (12). Additionally, Telli et al. reported on outcomes of lower pole stones < 10 mm in size managed with watchful waiting until stones increased in size or became symptomatic. In their cohort, ~66% required intervention; 33% percent were managed with watchful waiting. Of this former group, only 9% passed their stones spontaneously. Though these studies investigated conservative management of pediatric renal stones, their cohorts were limited by location of stones in the lower pole. These lower pole stones account for only 43% of stones in a pediatric patient cohort (13).

We sought to investigate the natural history of asymptomatic stones ≤5 mm in size in all renal locations from our institution. We hypothesized that no intervention would be required for the majority of asymptomatic, non-obstructing stones managed with watchful waiting.

## Methods

In this IRB-approved retrospective study, patients with a diagnosis of kidney stones from January 2011 to March 2017 were identified using ICD9 and ICD10 codes. On manual chart review we identified patients meeting the designated study criteria. Inclusion criterial included non-obstructive kidney stones 5 mm or less in size, bilateral, or unilateral. Patients with multiple renal non-obstructive stones that were 5 mm or less in size were included, and the size of the largest stone was noted for analysis. We also included patients with a history or prior stones and even prior interventions for stones. Date of initial imaging, usually renal ultrasound, identifying the non-obstructive stone was considered the date of diagnosis. Patients with multiple renal non-obstructive stones that were 5 mm or less in size were included, and the size of the largest stone was noted for analysis. Patients with a concomitant ureteral stone or obstruction were excluded, along with those that did not follow-up after initial imaging studies.

For all study patients, basic demographics (age, gender, ethnicity, comorbidities) were collected as well as stone specific history. For observational management of the stone, frequency of ultrasound imaging was noted, in addition to any stone-related ER visits, stone passage episodes, urinary tract infections and surgical interventions. For patients with a single febrile UTI, DMSA scan was not routinely performed. Time to stone passage, time to surgical intervention and total follow-up time were also documented. For patients undergoing surgical intervention, indication for surgery, operative details and outcomes were noted. Stone size and management at time of last follow-up were noted.

The medical management of these stones was also evaluated. We identified which patients were seen in a formalized stone clinic. Patients that had 24-h urine metabolic workup with Litholink were identified. Dietary modifications were defined as specific changes such as low salt diet or increased citrate. Increased fluid intake alone was not considered a dietary modification. Any long-term medications to address urine chemistry abnormalities were also recorded.

Data were analyzed using IBM SPSS Statistics 24 package. Patients with successful stone passage were compared to those who did not pass stones using Fisher's exact tests and Mann-Whitney tests. Kaplan Meier curves for stone formation were made to delineate and compare the natural history of such stone.

## Results

From 2011 to 2017, a total of 106 patients (41M, 65F) presented with non-obstructive kidney stones that were 5mm or less in size. The average age at initial presentation was 12.5 ± 5.5 years. 24 patients had bilateral kidney stones at diagnosis, while 44 had solely right sided kidney stones and 38 patients had solely left-sided kidney stones, amounting to a total of 130 renal unites (68 R, 62 L). The average stone size was 3.4 ± 1.1 mm and 49/130 (38%) had multiple stones. Average follow-up time was 17 months.

**Figure 1:**
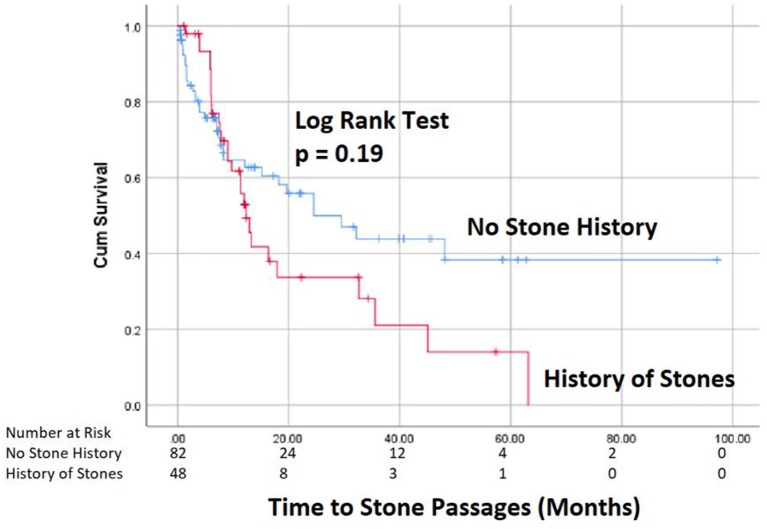
Kaplan Meier Curve for stone passage.

Spontaneous passage of stones occurred in 51% of patients, accounting for 47% (61/130) of the renal units at an average time of 11 months after diagnosis (Figure 1). Only 6/106 (5.7%) patients required stone surgery, all for unilateral stone disease with ureteroscopy (*n* = 4) or PCNL (*n* = 2) at an average time of 12 months after initial diagnosis. The indication for surgery in all 6 cases was pain. Two of these 6 patients passed their initial stone spontaneously, but later developed additional stones for which they required surgery secondary to pain. Febrile UTIs developed in 17/106 (16%) patients, 5 males, and 12 females, of which 2 patients had surgical interventions and 6 patients had spontaneous stone passage. Most patients (13/17) had a single febrile urinary tract infection. A total of 43 ER visits for stone-related issues were noted, but no patients required urgent intervention for an infected obstructing stone. Median time of follow-up was every 6 months with renal ultrasounds, which then was prolonged to annual follow up in most cases.

Table 1 compares patients that had spontaneous stone passage to those who did not have spontaneous passage. No significant differences were seen in basic demographics or urologic history between the two groups, although patients with a history of stones did tend to pass stones more frequently (46 vs. 29%, *p* = 0.07). Stone size, laterality or location was not significantly associated with successful stone passage (Table 2). Of the 10 renal pole stones, 70% had spontaneous passage, but due to a small sample size, this did not reach statistical significance. Stone passers did have a significantly higher frequency of ER visits (46 vs. 21%, *p* = 0.008), but no difference in occurrence of febrile UTIs. On univariate analysis of patients undergoing surgery, no associations were seen related to demographic variables, stone characteristics, ER visits, or history of febrile UTIs.

**Table 1:** Demographics, urologic history, and patient follow-up.

	**No Stone Passage (*n* = 52)**	**Spontaneous Passage (*n* = 54)**	***P*-value**
Average age (years)	13.3	11.8	*p* = 0.18
**Gender**			
Female	33 (63%)	32 (59%)	*p* = 0.69
Male	19 (37%)	22 (41%)	
**Ethnicity**			
Non-Hispanic White	26 (50%)	29 (54%)	*p* = 0.85
Non-Hispanic Black	2 (4%)	1 (2%)	
Hispanic	21 (40%)	20 (37%)	
Asian	1 (2%)	1 (2%)	
Unknown	2 (4%)	3 (6%)	
History of stones	15 (29%)	25 (46%)	*p* = 0.07
Previous stone surgeries	4 (8%)	7 (13%)	*p* = 0.29
Febrile UTIs	11 (21%)	6 (11%)	*p* = 0.19
ER Visits	11 (21%)	25 (46%)	*p* = 0.008
Frequency of follow-up	6.6 months	5.9 months	*p* = 0.23
Duration of follow-up	20.2 months	14.3 months	*p* = 0.08

**Table 2:** Stone characteristics for individual renal units.

	**No Stone Passage (*n* = 69)**	**Spontaneous Passage (*n* = 61)**	***P*-value**
**Laterality**			
Right	34 (49%)	33 (54%)	*p* = 0.36
Left	35 (51%)	28 (46%)	
Stone size (mm)	3.4	3.4	*p* = 0.98
Multiple stones	25 (36%)	24 (39%)	*p* = 0.43
**Location within kidney**			
Renal pelvis	3 (4%)	7 (11%)	*p* = 0.12
Upper pole	9 (13%)	9 (15%)	
Mid pole	19 (28%)	6 (10%)	
Lower pole	27 (39%)	29 (48%)	
Multiple	9 (13%)	9 (15%)	
Unknown	2 (3%)	2 (3%)	

Medical management was also undertaken for many of the patients with non-obstructive stones with 30% (32/106) being seen in a formalized stone clinic. Metabolic workup with 24-h urine chemistry was done in 29/106 patients (27%) and was significantly more commonly performed in patients with a prior history of stones (48 vs. 11%, *p* < 0.001). The majority of patients (62%) were given dietary modifications, while 16% were prescribed chronic medications. At time of last follow-up 5 patients had a noticeable increase in stone size up to 7 mm in size, but all of these patients remained on observation with no plans for surgery.

## Discussion

In this study, we describe the natural history of stones sized 5 mm or less in a pediatric population. In contrast to prior studies, the location of the stone within the kidney was not restricted (1, 3, 9, 12, 14). This is important since up to 60% of pediatric stone patients present with stones in locations other than the lower pole (13).

In our cohort, 51% of patients spontaneously pass stones measuring 5 mm or less at an average of 13 months after initial diagnosis. Our passage rate and timeline are mirrored by results found in another report that found a 54% passage rate of lower pole asymptomatic stones < 5 mm in size (12). While the presence of a prior stone history and renal pelvis location tended to have a higher spontaneous passage rates, these correlations did not reach statistical significance. Therefore, in our cohort, we were not able to identify any clear predictors for stone passage. It is notable that the reported stone passage rates have increased with higher rates reported in more recent studies (6, 15). This increase in stone passage rates may be related to the higher rapidly increasing incidence of kidney stones in younger age groups as well as the inclusion of smaller stone sizes that have higher spontaneous passage rates (16).

Our study was unique in that the location of the stone was expanded to all poles of the kidney to better reflect the pediatric stone patient. The most common location at stone presentation was the lower pole, but the stones in the renal pelvis had the highest rate of spontaneous passage. Based on renal anatomy it is intuitive to assume that lower pole stones should have the lowest passage rates, but in our population the difference did not reach statistical significance, likely due to underpowering. This is the first pediatric study that reports outcomes of watchful waiting of renal stones stratified by intrarenal location of pediatric stones. According to Koh et al., based on an adult cohort with an average stone size of 5.7 mm, highest stone passage rates occurred from stones originally presenting in the mid-pole (13). Further pediatric studies are needed to better delineate the difference in stone passage for different intrarenal stone locations.

Medical expulsive therapy (MET) has been described as useful for ureteral stone passage in adults (17–20). While there is moderate pediatric data for use of MET in ureteral stones, there is no data supporting the use of MET in pediatric nephrolithiasis (21). Similarly, in our patients, for the management of the non-obstructive kidney stones, MET was not prescribed. However, if patients presented with renal colic symptoms to the ER with active passage of their stone in the ureter, they were placed temporarily on MET to assist with stone passage. Since this was not controlled or studied directly in our cohort, it is not possible to deduce whether or not the use of MET assisted in spontaneous stone passage. Based on results from our study and those of Dos Santos, pediatric nephroliths ≤5 mm should not require empiric MET while stones are noted in the kidney since 51% spontaneously pass and another 23–43% did not require intervention.

It is notable that none of the ER visits for stone passage led to operative intervention due to failed stone passage. In fact, for the entire cohort, only 6/106 (6%) patients required surgical intervention for unilateral stone disease, all electively on an outpatient basis due to flank pain. This lower rate was likely due to the 5 mm stone size cut-off. Prior reports of larger lower pole stones suggest that stone size >7 mm was associated with surgical intervention (9).

At our institution, patients with kidney stones are followed annually at a minimum with a renal ultrasound. Based on our results, this schedule may not be necessary. Since most patients with kidney stones < 5 mm do not require intervention and those who required intervention did so for renal colic, it might be reasonable to forgo annual follow-up of patients with a single kidney stone < 5 mm in size. This management strategy would require further testing but could potentially relate a large annual cost-saving. A recent report on the financial burden of pediatric stone disease suggested that nephrolithiasis is on the rise compared to urolithiasis and management has shifted to the outpatient sector (22). Unfortunately, there are no financial data on solely outpatient nephrolithiasis management as recent reports focus on inpatient stone costs (22). Over 10 years, annual cost of nephrolithiasis in South Carolina rose $9.2 million to $12.6 million in 2007 (23). With further extrapolation to 2018 and to the whole of the United States, the potential cost savings is substantial.

On the other hand, recurrent stone formers need a greater focus on medical stone management with metabolic work-up, dietary changes and sometimes medical management. In our cohort, 40/106 (38%) patients had a history of stones and 30/106 (29%) were seen in a formalized stone clinic. The management of kidney stones in this group can focus on medical management rather than periodic renal ultrasounds to evaluate stone burden, since even in these patients, the only indication for surgery was renal colic. Therefore, additional imaging can be reserved for cases of symptomatic nephrolithiasis, be it pain or infection.

There are several strengths and weaknesses of our study. The 6-year time-period of the study was a strength, permitting long-term follow-up of the natural history of small non-obstructive renal stones. Furthermore, the inclusion of patients with previous stones, multiple stones and variable intrarenal locations allowed for a more representative cohort that can be potentially be extrapolated to other pediatric nephrolithiasis populations. While the use of renal ultrasound for diagnosis of renal stones limited our specificity and sensitivity in diagnosing kidney stones, it was preferentially used in children to avoid unnecessary radiation exposure. While our sample size included over 100 patients, the population is still relatively small when considering that only 6 patients required surgery and thus it is not possible to draw significant conclusions about risk factors for surgery. The sample size may also limit the power of our study in assessing predictors for spontaneous stone passage. Furthermore, the retrospective design of our study carries inherent biases with concern for variability in treatment strategies and poor follow-up in patients who may have been treated elsewhere or have resolution of symptoms. Due to this institution being a pediatric tertiary care center, it is likely that if there were any significant stone complications, they would be seen at our center. However, it is not reasonable to make such an assumption and thus our conclusion that there were no urgent intervention or complications of observing stones still can be questionable. Furthermore, due to our limited follow-up it is difficult to truly conclude that surveillance imaging is not necessary. Instead, our study merely suggest that this must be studied further with prospective evaluations to allow for complete understanding of watchful waiting in non-obstructive nephrolithiasis patients. Lastly, this study cannot be extrapolated to patients with renal stones >5 mm, which will need to be evaluated in future investigations.

## Conclusions

Based on our study cohort, we note a 47% spontaneous passage rate in non-obstructive renal stones ≤5 mm. No patients required urgent interventions for infected and/or obstructing ureteral stones while on observation, indicating that observation is a safe option in these patients. Furthermore, the few patients that required intervention presented with renal colic; therefore, this study begins to question the need for repeated imaging surveillance, and rather encourages a bigger focus on medical stone management in recurrent stone formers.

## Ethics statement

Institutional Review Board approved the study.

## Author contributions

KS, AS, and SR conceptualized the study with significant clinical input from AM, CJ, CK, DR, DT, EG, NJ, and PA. KS, AP-O, ND, CH, and CG performed retrospective patient chart review. KS, JW, and AS interpreted data, performed statistical analyses and drafted the manuscript. KS, JW, and AS performed multiple revisions of manuscript. All authors revised, read, and approved the final manuscript.

### Conflict of interest statement

The authors declare that the research was conducted in the absence of any commercial or financial relationships that could be construed as a potential conflict of interest.

## Abbreviations

ER: Emergency Room
IRB: Institutional Review Board
ICD: International Classification of Diseases
MET: Medical Expusive Therapy
PCNL: Percutaneous Nephrolithotomy
UTI: Urinary Tract Infection.
